# A Review of Episiotomy and Modalities for Relief of Episiotomy Pain

**DOI:** 10.7759/cureus.31620

**Published:** 2022-11-17

**Authors:** Rutuja G Choudhari, Surekha A Tayade, Shreya V Venurkar, Vaishnavi P Deshpande

**Affiliations:** 1 Obstetrics and Gynaecology, Jawaharlal Nehru Medical College, Datta Meghe Institute of Medical Sciences, Wardha, IND

**Keywords:** postnatal mother, sitz bath, infrared lamp therapy, wound healing, episiotomy

## Abstract

Episiotomy is a surgically planned incision of the perineum and the posterior vaginal wall in the second stage of labour. It is one of the most commonly performed surgical procedures in the world. In episiotomy, the vaginal orifice is made larger to facilitate the birth of a baby. The postnatal period is very crucial in every mother's life, especially those who had a vaginal delivery and underwent episiotomy. Maternal benefits of episiotomy include reduced risk of perineal trauma, subsequent pelvic floor dysfunction and prolapse, urinary incontinence, faecal incontinence, and sexual dysfunction. Potential benefits for the foetus are thought to include a shortened second stage of labour. However, an episiotomy can also lead to potential adverse consequences, including the extension to a third- or fourth-degree tear, anal sphincter dysfunction, and dyspareunia. Different approaches can be adopted to reduce these complications in the postpartum period, including cleanliness, cold packs, sitz baths, kegel exercises, perineal care, and topical application of dry heat-infrared lamp therapy. Of all these approaches, infrared lamp therapy and sitz baths are the two most effective and commonly used for episiotomy pain relief and wound healing. In infrared lamp therapy, radiant heat or infrared rays are used to produce heat that is then applied to the episiotomy wound to facilitate pain relief and wound healing, while a sitz bath is a moist heat application process that is also effective for episiotomy pain relief and wound healing. This review aims to offer the most thorough understanding of episiotomy, its current concept, and episiotomy pain relief, with a particular focus on infrared lamp therapy and sitz baths.

## Introduction and background

Giving birth is a life-changing event in a woman’s life and has a long-lasting impact on her life, both physically and mentally [1]. It is the most joyful experience for her and her entire family, but at the same time, several complications in the postnatal stage, including perineal pain, constipation, breast engorgement, cracked nipples, fatigue, backache, and headache, can cause the mother severe physical and psychological distress and reduce her quality of life [2]. Mothers may also face discomfort due to the physiological process of uterine involution in the postnatal stage. Because mothers have to go through all these complications simultaneously, they need extra care. Annually, 120,243 vaginal births take place in India, with 63.4% of them having an episiotomy. Episiotomy rates in primiparous women are 8.8 times higher than those in multiparous women [3]. The World Health Organization (WHO) reported that there are 500,000 maternal deaths per year, of which 99% occur in developing countries [4].

The American College of Obstetrics and Gynecology estimates that one in three women who have a vaginal delivery also undergo an episiotomy [5]. The episiotomy rates vary widely across countries depending on their restrictive or routine use. As reported in the literature, episiotomy rates range from 8% in the Netherlands, to 13% in England, to 25% in the US. Episiotomy rates are still high in developing nations because primigravidae have not been largely adopted in those nations, limiting the use of episiotomy [6]. Episiotomies in the US have declined since the late 1970s, from 61% in 1979 to 25% in 2004 [7]. Perineal pain and discomfort, episiotomy infections, and puerperal sepsis are all sources of morbidity and mortality in women in the postnatal stage. Perineal pain and discomfort are one of the leading contributors to maternal morbidity. A study reported that 1,345 Nigerian women who gave vaginal births also had episiotomies. Over 90% of primigravidae had episiotomies, while the prevalence of episiotomies was 46.6% [8].

## Review

Episiotomy

**Figure 1:**
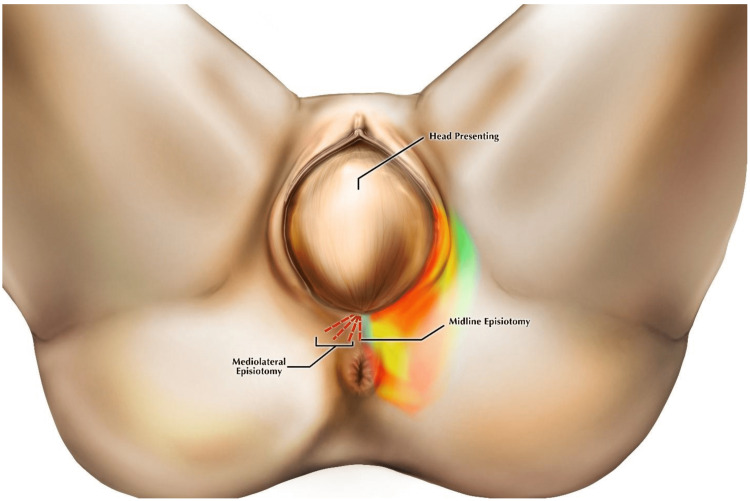
Mediolateral and midline episiotomy incision over the perineum. Open access journal under a CC-BY license. Contributed by Garner et al. [10].

Episiotomy, also known as perineotomy, is a planned surgical incision of the perineum to augment the second stage of labour. In the second stage of labour, when the crowning of the baby occurs, that is, when the presenting part of the head of the baby is visible, an episiotomy is performed to widen the gap and expedite labour. In the second stage of delivery, the perineum and posterior wall of the vagina is surgically incised to promptly widen the gap. It enables the baby to pass through the route without causing discomfort to the mother or the baby [9]. Different types of episiotomy are performed depending on where the incision needs to be made, including median, J-shaped, and mediolateral episiotomy and lateral episiotomy. Among these different types of episiotomy, the mediolateral type is the most prevalent. However, the WHO does not recommend episiotomy for every woman undergoing normal vaginal delivery. The difference between median episiotomy and mediolateral episiotomy is depicted in Table 1. Figure 1 depicts the mediolateral and midline incision for episiotomy [10].

**Table 1:** Difference between median episiotomy and mediolateral episiotomy. The authors created this table.

Median or midline episiotomy	Mediolateral episiotomy
The incision is made vertically over the perineum	The incision is made at an angle over the perineum
It is equivalent to a first-degree perineal tear	It is equivalent to a second-degree perineal tear
Fewer muscle fibres are cut, causing less bleeding	More muscle fibres are cut, causing relatively more risk of bleeding
If episiotomy extends, it can involve the anal sphincter and can lead to faecal incontinence	It does not involve the anal sphincter, and if necessary, the incision can be extended
Dyspareunia is rare	Dyspareunia is common
Repair is easy, and healing is prompt	Repair is difficult, and healing is delayed
Post-operative comfort is maximum	Post-operative comfort is relatively lesser

Some specific consequences of episiotomy are foetal distress, premature delivery, shoulder dystocia, after-coming head in breech births, instrumental delivery, macrosomia, and face-to-pubis delivery. Structures cut during episiotomy are skin, subcutaneous tissue, superficial and deep perineal fascia, superficial and deep transverse perineal muscles, bulbospongiosus, part of the levator ani, transverse perineal branches of pudendal vessels and nerves, and posterior vaginal wall, in this order. Muscles that are not cut during episiotomy are ischiococcygeus, ischiocavernosus, and anal sphincters. Significant complications are also associated with episiotomy, such as pain, oedema, hematoma, infection, and, ultimately, dyspareunia brought on by a sensitive vaginal scar or a narrowed vagina [11]. In an episiotomy, an incision is made along the perineum, which is a sensitive area. The muscles in the perineum are involved in daily actions such as sitting, walking, bending down, squatting, urination, and excretion. An incision on the perineum results in pain and may cause discomfort to postnatal women in performing these daily activities [12]. It is usually performed during vaginal delivery for childbirth [13].

The following are some of the benefits of episiotomy: lower incidence of posterior perineal trauma, fewer sutures, and quicker recovery than tears. Perineal tears, particularly third-grade perineal cuts, should be avoided by making clean incisions adjacent to them. They are more likely to keep the pelvic floor and perineum muscles relaxed, which will improve sexual functioning and reduce the risk of faecal and urine incontinence in postnatal women. The need for sutures and postpartum healing difficulties such as loss of blood, oedema, haematoma, infections, wound dehiscence, and perineal pain is associated with episiotomy, a standard obstetric procedure [14]. Few patients endure postpartum pain and discomfort, which can linger well past the puerperium as long-term pain and dyspareunia. Furthermore, infections, wound breakdown, urine and faecal incontinence, and other side effects of perineal trauma treatment could make the postpartum period exceedingly tricky [15]. Postpartum pain can be assessed by the redness, oedema, ecchymosis, discharge, and approximation scale. Sexual dysfunction after delivery is frequently caused by postpartum pain due to tears in the perineum during an episiotomy [16].

Some critical problems linked with episiotomy are pain, oedema, haematoma, infection, and, ultimately, dyspareunia caused by a painful vaginal scar or vaginal constriction. It may result in discomfort or impaired sexual functioning for up to a year following a perineal injury that is very deep or numerous. Patients who underwent an episiotomy or suffered a perineal tear during delivery are more likely to continue to engage in sexual activities. Compared to women who have not undergone these treatments, those who have could feel more pain during a sexual act [17]. A cross-sectional study found that 67% of women did not seek special care six weeks to six months after giving birth [18]. This study also found that postpartum dyspareunia was more common in primiparous women. As a result, postpartum women frequently experience mental health issues, which might influence how they feel about their newborns [9].

To expedite episiotomy wound recovery, many procedures have been utilised. This method has been designed to relieve pain, facilitate comfort, and prevent episiotomy infection. In this method, the following procedures are implemented: cleanliness, cold packs, sitz baths, kegel exercises, perineal care, and topical application of dry heat-Infrared therapy whose effects last for an extended period, and keeps the wound dry [19]. This increases blood flow, reduces pain, and increases the amount of oxygen and nutrition available to the tissue. Additionally, it facilitates the resolution of inflammation, expedites the evacuation of waste, and promotes muscle relaxation. The release of the chemical vasodilator histamine also has physiological effects on cutaneous vasodilation and alleviates pain [20]. Some methods to speed healing and relieve discomfort include exposing the perineum to the air by allowing the pad down while napping or resting, avoiding positions that impose pressure on this area, such as long durations of standing or sitting, laying sideways while sleeping or napping, sitting while bathing or sitting in warm water in a tub, using ice packs made of gauze soaked in cold water to ease discomfort, and avoiding strains during defecation or urination. Women should seek medical help when the following occur: foul-smelling vaginal discharge, burning pain during urination, severe pain in any of the perineum, lower abdomen or pelvis, fever, and heavy vaginal bleeding [21].

Infrared therapy

**Figure 2:**
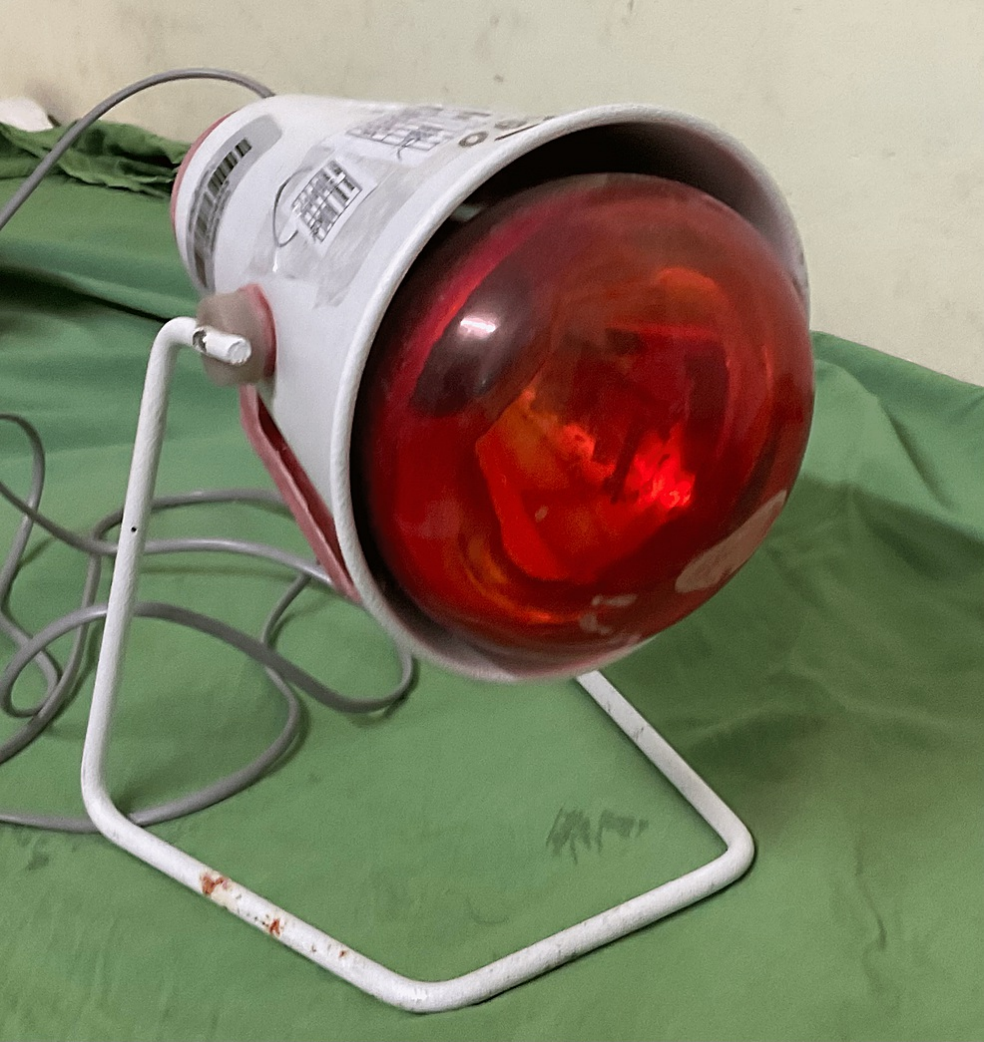
An infrared heat therapy lamp. The authors have captured this image.

Infrared radiation is electromagnetic radiation with wavelengths between 760 nm and 100,000 nm. Infrared therapy is a safe, drug-free, and effective way to reduce pain and inflammation throughout the body using light. Figure 2 shows an infrared heat therapy lamp.

Physiological effects of infrared therapy include the following: after one to two minutes, histamine, a chemical vasodilator, is released, possibly acting on blood vessels and, thus, causing local cutaneous vasodilation. The therapeutic uses of infrared therapy include pain relief (chronic back pain), acceleration of healing and tissue repair (pressure sore), decrease in muscle spasms, used before electrical stimulation/testing, and reduction of dangers, burns, skin irritation, dehydration, hypotension, and defective arterial blood flow. Contraindications for infrared lamp therapy usage are skin irritation, malignancy, fever, impaired cutaneous thermal sensation, and skin damage due to ionising radiation and defective arterial cutaneous circulation [22].

Infrared therapy is a one-of-a-kind treatment in which the healing impact of light is used for facilitating pain and discomfort relief and expediting the healing of episiotomy wounds. This therapy involves exposing the incision site or the diseased region of the perineum to infrared radiation from a light source of 230 volts from a distance of 45-50 cm for 10-15 minutes, which provides relief from discomfort. The treatment is straightforward, painless, and has no known adverse effects [23,24]. Infrared rays have a therapeutic impact by aggregating blood supply to a specific location and alleviating pain. This improves the tissue’s access to nutrients and oxygen while accelerating the expulsion of waste, which assists in the reduction of inflammation. Due to the cooling effect on the external sensory nerve terminals, the discomfort is virtually certainly diminished when the heat is mild. It can also relax the muscles and relieve muscle spasms caused by injuries [25]. For patients with episiotomy injuries, infrared therapy is an appropriate option. The production of chemical vasodilators, such as histamine and other similar substances, has a biological effect on cutaneous vasodilation and a potential direct effect on blood vessels [25,26]. Good blood circulation is essential for wound healing, preventing infection, and destroying bacteria, which is precipitated by dry heat therapy.

Sitz bath

Another important method that is commonly used for episiotomy wound healing is a sitz bath. For a sitz bath, a person is asked to sit in a tub filled with water to their hip level. A sitz bath is often referred to as a hip bath. The term "sitz bath" is derived from the German word *Sitzbad*, meaning a bath (*bad*) in which one sits (*sitzen*). A sitz bath can be administered using warm or chilly water. However, to relieve the pain, itching, and discomfort, it is suggested that warm water be used for a sitz bath [15,27]. The region between the rectum and the vulva, or scrotum, is known as the perineum, which is cleaned with a sitz bath, warm water, and a shallow bath [28]. Additionally, a sitz bath helps ease discomfort or itchiness in the vaginal area. In addition to preventing soreness and a burning sensation around the perineum, the benefits of using a sitz bath include minimising perineal irritation, inflammation, and swelling. Indications for using a sitz bath are haemorrhoids, anorectal infections, operations, and postpartum mothers who gave birth vaginally [29].

In a sitz bath, the perineal area and buttocks are submerged in warm water for 15-20 minutes at a temperature of 40-45°C. This may heal wounds and relieve pain, itching, or muscle spasms. A sitz bath may heal wounds by cleaning the perineum and the anus, increasing circulation, reducing oedema and inflammation, and promoting muscle relaxation [30]. Postnatal mothers might have painful micturition because of local bruises on the vulva, clitoris, and vagina and an episiotomy scar. Urine retention may occur due to a sore spot or the operative delivery. Sitz baths provide pain relief; infrared exposure relieves perineal pain. Analgesics may be required for treating local pain [31]. Table 2 depicts the difference between infrared lamp therapy and a sitz bath.

**Table 2:** Difference between infrared lamp therapy and a sitz bath. The authors created this table.

Infrared lamp therapy	Sitz bath
It is a dry heat method	It is a moist heat method
Radiant heat or infrared rays are used to apply heat to the episiotomy wound	A postnatal woman is asked to sit in a warm water tub up to the hip level for heating the episiotomy wound
An infrared lamp is placed at a distance of 45 cm from the perineum, and the heat is produced at 230 volts for 10 minutes	The woman is asked to sit in a basin (tub) filled with warm water (45-59℃), without pressure on the perineum, with her feet flat on the floor for 10 minutes
Advantage: It produces drying of the skin and softens the surrounding area, penetrates deep into the tissue layer, doesn’t induce sweating, and prevents fluid loss	Advantage: It has a low risk of skin burns, without causing skin maceration, and retains normal temperature, as evaporation does not occur in this process
Disadvantage: There is a greater risk of bleeding and a chance of skin irritation, malignancy, fever, impaired cutaneous thermal sensation, or skin damage due to ionising radiation	Disadvantage: It leads to increased body fluid loss through penetration, as it does not penetrate deep into the tissue, and can result in increased drying of the skin
Other uses of infrared lamp therapy: Reduction of psoriasis, diabetes-related complications such as diabetic foot ulcers, inflammation and pain from rheumatoid arthritis, healing of burns, amputation injuries, skin grafts, infected wounds, and trapping injuries	Other uses of a sitz bath: Pain relief and reduction of stiffness and secondary muscle spasm in chronic arthritis, acute temporomandibular joint-closed lock condition, and pain and muscle spasms on posterior neck and back in patients with ankylosing

Both procedures can be used and are effective in the prevention and fast healing of the wound. However, puerperal mothers who received infrared lamp therapy on their episiotomy wound experienced faster wound healing and less pain than those who took a warm sitz bath. The application of infrared lamp therapy has a significant effect on reducing episiotomy pain and promoting wound healing among postnatal mothers [32]. The primary goal of medical treatment in the modern era, when medical care and treatment expenses are increasing, is to offer affordable care to patients. If nurses and midwives recognise the importance of their care in episiotomy wound healing, they can provide therapies that are both effective and affordable. The greatest method to give consideration is to enable nurses and other medical professionals to adjust their routines [33].

Nurses and midwives play a vital role in the overall management of perineal pain and wound healing after episiotomy, including continuous pain assessment and wound healing evaluation, application of interventions for episiotomy wounds, instruction for the new mothers about perineal self-assessment and care, and communication of relevant information about pain and healing process that every postnatal mother have [24,34].

Between the pretest and post-test, a statistically significant difference was discovered. It was found that both dry and moist heat therapies were successful [35]. However, for postpartum women, dry heat therapy with a hair dryer proved to be more successful at reducing episiotomy discomfort compared to hot heat from a sitz bath [28]. The healing time after an episiotomy was shortened from 14 days to 7 days with this dry heat treatment. Therefore, postnatal women are advised to practise this method both in the hospital and after being discharged. Previously, for the maintenance of episiotomy, most heat applications such as sitz baths and hot packs were used. With scientific advancement, more dry heat therapies were developed such as electric heat lamps, peri lights, and infrared rays. According to research, dry heat is more effective than moist heat because its action lasts longer, keeps the area dry, and aids in wound healing [26].

## Conclusions

Episiotomy is the most commonly performed planned surgical incision on the perineum during the second stage of labour. Indications of episiotomy are foetal distress, complicated baby positions such as breeches, premature births, large babies, and vacuum delivery. Maternal benefits are reduced risk of perineal trauma, subsequent pelvic floor dysfunction and prolapse, urinary incontinence, faecal incontinence, and sexual dysfunction. Potential benefits for the foetus were thought to include a shortened second stage of labour as a result of more rapid and spontaneous delivery. It can also result in adverse consequences of episiotomy, including an extension to a third- or fourth-degree tear, anal sphincter dysfunction, and dyspareunia. Infrared lamp therapy is a one-of-a-kind treatment procedure where the healing impact of light is used to cure pain and discomfort and also expedite episiotomy wound healing. Heat waves cause cutaneous vasodilation, as well as a potential direct influence on blood vessels, which is very effective in pain relief and wound healing. The other method considered in this review is the administration of moist heat using sitz bath therapy. Special indications of performing infrared lamp therapy over a sitz bath include the following: it penetrates heat deep into the tissue layer, doesn’t induce sweating, and prevents fluid loss. Based on the findings in most articles cited in this review, we conclude that infrared lamp therapy is a more effective method for postnatal episiotomy pain and wound healing among postnatal mothers compared to moist heat therapy with a sitz bath. Hence, infrared lamp therapy should be included in the hospital routine for better management of daily care for postpartum mothers with episiotomy wounds. Nurses and midwives play a vital role in the overall management of perineal pain and wound healing after episiotomy, so they should be educated about performing this dry heat therapy.
